# Production of Recombinant Monoclonal Antibodies in the Egg White of Gene-Targeted Transgenic Chickens

**DOI:** 10.3390/genes12010038

**Published:** 2020-12-30

**Authors:** Takehiro Mukae, Sho Okumura, Takuma Watanobe, Kyoko Yoshii, Takahiro Tagami, Isao Oishi

**Affiliations:** 1 Biomedical Research Institute, National Institute of Advanced Industrial Science and Technology, 1-8-31, Ikeda, Osaka 563-8577, Japan; t-mukae@aist.go.jp (T.M.); j214ky-yoshii@aist.go.jp (K.Y.); 2 Sapporo Division, Cosmo Bio Co. Ltd., 3-513-2, Zenibako, Otaru, Hokkaido 047-0261, Japan; sho-okumura@cosmobio.co.jp (S.O.); takuma-watanobe@cosmobio.co.jp (T.W.); 3 Animal Breeding and Reproduction Research Division, Institute of Livestock and Grassland Science, National Agriculture and Food Research Organization, 2 Ikenodai, Tsukuba, Ibaraki 305-0901, Japan; tagami@affrc.go.jp

**Keywords:** chicken, knock-in, genome editing, bioreactor, antibody production

## Abstract

Increased commercial demand for monoclonal antibodies (mAbs) has resulted in the urgent need to establish efficient production systems. We previously developed a transgenic chicken bioreactor system that effectively produced human cytokines in egg whites using genome-edited transgenic chickens. Here, we describe the application of this system to mAb production. The genes encoding the heavy and light chains of humanized anti-HER2 mAb, linked by a 2A peptide sequence, were integrated into the chicken ovalbumin gene locus using a CRISPR/Cas9 protocol. The knock-in hens produced a fully assembled humanized mAb in their eggs. The mAb expression level in the egg white was 1.4–1.9 mg/mL, as determined by ELISA. Furthermore, the antigen binding affinity of the anti-HER2 mAb obtained was estimated to be equal to that of the therapeutic anti-HER2 mAb (trastuzumab). In addition, antigen-specific binding by the egg white mAb was demonstrated by immunofluorescence against HER2-positive and -negative cells. These results indicate that the chicken bioreactor system can efficiently produce mAbs with antigen binding capacity and can serve as an alternative production system for commercial mAbs.

## 1. Introduction

Monoclonal antibodies (mAbs) are the most effective class of biopharmaceuticals for the treatment of diseases [1,2]. In fact, the market for therapeutic mAbs has significantly increased over the past few decades [3]. In addition to therapeutic mAbs currently in use, numerous mAbs are being developed and tested in clinical trials [4]. Therefore, the demand for their production is predicted to continuously increase going forward. Presently, most therapeutic mAbs are produced as recombinant proteins using mammalian cultured cells [5]. However, this approach requires expensive production facilities and complex production control. The high manufacturing costs of therapeutic mAbs creates significant economic and social burdens; therefore, the development of a low-cost alternative technology is imperative for the continued use and development of this class of biopharmaceuticals. Alternative approaches include the development of bioreactors using transgenic plants, insects, and mammals [5,6,7]. Such transgenic organisms do not require expensive facilities and can produce foreign proteins in their products including the plant body, cocoon, and milk, at low cost [8,9]. Therefore, they have great potential as alternative methods of mAb production that reduce associated costs and increase efficiency.

Transgenic chickens, which produce functional mAbs in their eggs, provide a useful bioreactor for cost-effective mAb manufacture [10]. Furthermore, a transgenic chicken bioreactor has several advantages over other transgenic organisms, including short generation time, facile flock expansion, human-like glycosylation profiles, low housing costs, and simple containment requirements [11]. In 2005, the production of human mAbs in egg whites, using transgenic chimera chickens, was first reported [12]. In this study, chicken embryonic stem (ES) cells were transfected with DNA vectors designed to specifically express human mAbs in chicken oviduct magnum, and chimera chickens were established via transplantation of the ES cells to recipient embryos. The resulting chimera hens produced 34–148 µg/mL of mAbs in their egg whites; however, transgenic offspring were not obtained from this study. Recently, heritable transgenic chickens capable of producing mAbs in their egg whites were generated using chicken primordial germ cells (PGCs) [13]. In this study, a gene construct, designed to expresses the mAb in chicken oviduct magnum, was randomly integrated into the genome of the chickens. Functional biosimilar mAbs were produced in the egg white of the transgenic hens; however, expression levels were limited to 2–18 µg/mL. The low production efficiency of ectopic proteins in transgenic chickens can be attributed to gene silencing or the so-called positional effect of transgenes [14,15]. Therefore, it is critical that the transgene integrates into the host chicken genome in such a way that avoids translational suppression [10]. 

The use of lentiviral vectors is a promising method to avoid silencing of transgenes in a transgenic chicken bioreactor [16,17]. However, the packaging limit of the vector could be an obstacle in the introduction of large-size transgenes, which allows the abundant expression of mAbs in the chicken oviduct magnum. 

Recently, we developed transgenic chickens that efficiently produced a foreign protein in their egg whites [18]. In this study, the gene encoding human interferon β (IFN-β) was integrated into a major egg white gene, the ovalbumin gene (*OVA*) locus, of PGCs using a CRISPR/Cas9 system (gene knock-in; KI). The KI chickens were then established using the modified PGCs and were found to abundantly produce recombinant human IFN-β in their egg whites (1.9–4.4 mg/mL). Thus, we anticipated abundant mAb production in egg whites using the same approach to generate KI chickens via insertion of mAb genes at the *OVA* locus. 

The purpose of this study was to examine whether mAbs could efficiently be produced in the egg white of a KI chicken. We integrated the genes encoding humanized anti-HER2 antibody at the initiation site of the *OVA* locus of PGCs to establish KI hens. We analyzed the mAbs in the eggs laid by the KI hens and compared the binding affinity and selectivity with those of trastuzumab, a commercially available therapeutic anti-HER2 antibody.

## 2. Materials and Methods

### 2.1. Animal Experiments

All animal experiments were conducted according to protocols approved by the Institutional Animal Care and Use Committees of the National Institute of Advanced Industrial Science and Technology Tsukuba, Japan (protocol number 2016-115), the Institute of Livestock and Grassland Science (NILGS), National Agriculture and Food Research Organization (NARO), Tsukuba, Japan (protocol number 1611B056), and Cosmo Bio Co. Ltd., Tokyo, Japan, (protocol number PMC201703). Knock-in (KI) and wildtype (WT) chickens were maintained and bred at the animal farm facilities of NARO-NILGS and Cosmo Bio Co. Ltd.

### 2.2. Plasmid Construction 

The plasmid expressing hCas9 and single-guide RNA (sgRNA) targeted to OVA (px330-Neo-OVATg2) were generated as described elsewhere [19]. In the current experiment, the puromycin resistance gene was inserted instead of the neomycin resistance gene in the plasmid (px330-Puro-OVATg2). The donor construct for the mAbs was generated by ligating PCR fragments consisting of 2.8 kb of *OVA* DNA (upstream of the ATG initiation codon) as the 5′ homology arm, complementary DNA (cDNA) encoding the heavy and light chains of the anti-HER2 mAb with the lysozyme signal sequence at their respective 5′-ends and linked through the sequence encoding the furin-2A peptide, the bovine growth hormone polyadenylation (bGH-pA) sequence, neomycin resistance gene sequence, and 3.2 kb of the *OVA* DNA sequence as the 3′ homology arm (Figure S1, Supplementary Materials). The donor construct was cloned into the *Sal*I and *BamH*I sites of the pBluescript II SK(+) vector plasmid (Takara Bio, Mountain View, CA, USA) and named the pBS-mAb donor.

### 2.3. Generation of Knock-In Hens 

The KI chickens were generated using essentially the same method as described previously [16]. Briefly, px330-Puro-OVATg2 and pBS-mAb donor were cotransfected into cultured chicken primordial germ cells (PGCs) and selected with 0.5 mg/mL neomycin for 5 days, following 2 days of culture growth with 1 µg/mL puromycin. The selected cells were proliferated and transplanted into the bloodstream of the recipient chicken embryo to generate germline chimera chickens. A chimeric rooster was crossed with a wildtype hen, and mAb KI offspring were identified by PCR genotyping using MightyAmp DNA Polymerase Ver. 2 (Takara Bio, Kusatsu, Japan). Thermocycling conditions were as follows: 98 °C for 2 min, 35 cycles of 98 °C for 10 s, 60 °C for 3 min, and 72 °C for 30 s. Sequences of primers used in this study were as follows: P1: 5′–ACCTGTGGTGTAGACATCCAGCA–3′, P2: 5′–CCCCAGAGCAGCCAGGGGCAGGAAGCAAAG–3′, P3: 5′–GCACTCTGTCGATACCCCACCGA–3′, P4: 5′–CAACTTCTAGGGCCATACCTGCT–3′, P5: 5′–AAAATTCCATGCTTGCTGCACCGAT–3′. Eggs from two 10 month old KI hens (#41 and #42) were used in this study.

### 2.4. Preparation of WT and KI Egg White 

A disposable medical dropper was used to collect KI and WT egg white samples. The egg whites were homogenized through sonication using an ultrasonic homogenizer (VP050N, TAITEC, Koshigaya, Japan). A portion of each sample was suspended in phosphate-buffered saline (PBS) and appropriately diluted.

### 2.5. Antibodies

Trastuzumab (Chugai, Tokyo, Japan) was purchased and diluted in PBS. Anti-human immunoglobulin G (hIgG) Fc (I-124, Jackson ImmunoResearch, West Grove, PA, USA), anti-hIgG heavy and light chains (hIgG (H + L), AB_2337577, Jackson ImmunoResearch), and Alexa Fluor goat anti-mouse IgG (H + L) antibodies (Alexa Fluor, A-11029, Thermo Fisher Scientific, Waltham, MA, USA) were also purchased.

### 2.6. SDS-PAGE and CBB Staining

Egg white samples from WT and mAb KI chickens were suspended in PBS at a ratio of 1:10 for SDS-PAGE. Samples were mixed with an equal volume of 2× Laemmli SDS-PAGE buffer (0.125 M Tris-HCl, pH 6.8, 10% (*w/v*) sucrose, 4% (*w/v*) SDS, 10% (*v/v*) 2-mercaptoethanol, and 0.01% bromophenol blue) and 10 µL of each was subjected to SDS-PAGE (5–20% *w/v* acrylamide; Oriental Instruments Ltd., Sagamihara, Japan). Samples were prepared for nonreducing SDS-PAGE analysis using 2-mercaptoethanol-free SDS-PAGE sample buffer. The gels were stained with Coomassie Brilliant Blue (CBB) R-250 solution (Nacalai, Kyoto, Japan). Precision Plus Protein Standard (BIO-RAD, Hercules, CA, USA) was used as a protein molecular weight marker.

### 2.7. Immunoblot Analysis

For immunoblot analysis, WT and mAb KI egg white samples were suspended in PBS at a ratio of 1:100 and dissolved in an equal volume of 2× Laemmli SDS-PAGE buffer, with or without 2-mercaptoethanol. Samples (10 µL each) were separated by SDS-PAGE, and then transferred onto a polyvinylidene difluoride membrane (Immobilon-P; Millipore, Bedford, MA, USA) via electroblotting. After blocking with 1% bovine serum albumin in 10 mM Tris-HCl containing 150 mM NaCl and 0.1% Tween-20 (TBS-T), the membrane was subjected to immunoblotting with antibodies against hIgG Fc or hIgG (H + L) at a 6000-fold dilution. Proteins were visualized with horseradish peroxidase-conjugated anti-mouse (for hIgG Fc) (Jackson ImmunoResearch) and anti-rabbit IgG (for hIgG (H + L)) (Jackson ImmunoResearch) using an enhanced chemiluminescence system (ImmunoStar reagent; Wako, Osaka, Japan). The immunoreactive bands were analyzed with ImageQuant LAS 500 (GE Healthcare, Wauwatosa, WI, USA) and the relative intensity of the bands was determined using the ImageJ software (National Institutes of Health, Bethesda, MD, USA). 

### 2.8. Enzyme-Linked Immunosorbent Assay (ELISA)

The concentration of hIgG in KI egg white was determined using human immunoglobulin G (IgG) AssayMax ELISA kit from AssayPro (St. Charles, MO, USA), according to the protocols provided by the manufacturer. The HER2 antigen binding activity of mAbs in KI egg white was evaluated using a Herceptin ELISA kit (α diagnostic International San Antonio, TX, USA), with commercial trastuzumab as a reference. The color generated in each ELISA was quantified using a microplate reader (iMark, BIO-RAD).

### 2.9. Immunofluorescence

Glass slides mounted with 4–5 μm thick slices of formalin-fixed paraffin-embedded human HER2-positive and -negative cells (POSICON-slide HER2 IHC, Pathology Institute, Toyama, Japan) were purchased and used for immunofluorescence. The sections on the glass slides were deparaffinized and rehydrated sequentially with xylene, ethanol, and water. The sections were treated with blocking solution (Blocking ONE histo, Nacalai) for 30 min, and then incubated with 1 µg/mL anti-HER2 antibodies (diluted KI egg white or trastuzumab) for 30 min at room temperature. After a secondary antibody reaction with mouse anti-hIgG Fc (1:1000, I-124) for 30 min, sections were visualized with Alexa Fluor 488 goat anti-mouse IgG (H + L) antibody (1:1000). The sections were mounted using Fluoroshield with DAPI (H-1200, ImmunoBioScience Corp, Mukilteo, WA, USA), and images were obtained using a fluorescence microscope (BZ-800; Keyence, Osaka, Japan).

## 3. Results

### 3.1. Generation of Anti-HER2 mAb KI Chickens

**Figure 1:**
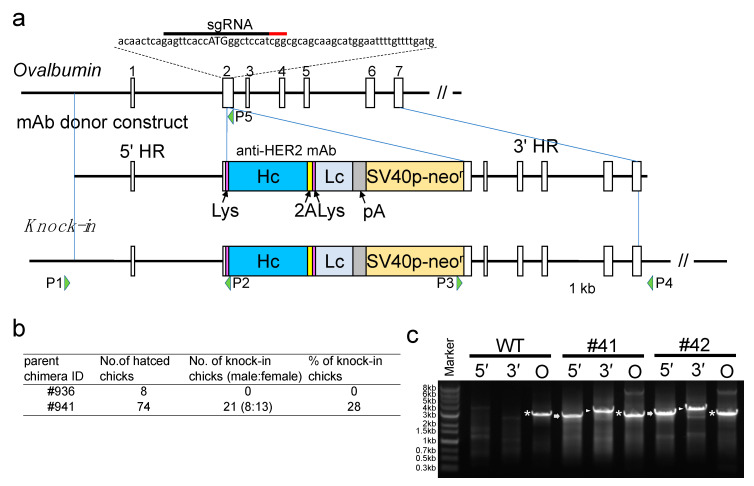
CRISPR/Cas9-mediated humanized anti-HER2 monoclonal antibody (mAb) gene knock-in at the initiation site of the *OVA* locus. (**a**) Schematic representation of the knock-in for the *OVA* locus. The top diagram shows exon–intron organization for the *OVA* locus. Boxes with numbers indicate *OVA* exons. The target single-guide RNA (sgRNA) and the protospacer adjacent-motif sequences in exon 2 are indicated by the black and red bars, respectively, above the nucleotide sequence. The initiation codon of *OVA* is shown in capital letters. The middle diagram shows the mAb donor construct. It consists of a 5′ homology region (HR), followed by DNA sequences of the lysozyme signal (Lys), heavy chain of the anti-HER2 mAb (Hc), furin-2A peptide (2A), another Lys, light chain of the anti-HER2 mAb (Lc), bovine growth hormone polyadenylation signal (pA), neomycin resistance gene driven by the SV40 promoter (SV40p-neo^r^), and a 3′ HR. The bottom diagram shows the knock-in (KI) allele. P1 to P4 indicate the primers used for 5′ and 3′ *OVA* assays. (**b**) Percentage of KI chicks among the offspring of chimeras. (**c**) PCR amplification of the KI allele in the genome of KI chickens. The genomes of mAb KI (#41 and #42) and wildtype (WT) hens were PCR amplified using primers P1–P2, P3–P4, and P1–P5 for 5′, 3′, and endogenous *OVA* assays, respectively. PCR amplicons, showing the expected sizes of 2.8 kb for the 5′ assay (5′), 3.3 kb for the 3′ assay (3′), and 2.8 kb for the endogenous *OVA* assay (O), are indicated by the arrows, arrowheads, and asterisks, respectively.

To generate KI chickens carrying the gene encoding the anti-HER2 mAb at the initiation site of the *OVA* locus, we prepared pX330-Puro-OVATg2, as an sgRNA/Cas9 (sg, single guide RNA) plasmid, and pBS-mAb donor, as a donor plasmid (Figure 1a). The donor construct contained the DNA sequence of heavy and light chains of the anti-HER2 mAb linked through the sequence encoding the furin-2A peptide; thus, the expression of the anti-HER2 full antibody (H2L2) was expected in the egg white of the generated KI chickens. Chicken PGCs were cotransfected with these plasmids, selected with antibiotics, and transplanted to the recipient embryos to generate germline chimera. Two presumptive chimeric roosters were raised and crossed with WT hens. The genotype of the progeny was assessed by PCR, and 28% of the progeny from one rooster (#941) were identified as KI chickens (Figure 1b,c). All the KI chickens had normal appearance and no distinguishable growth defects.

### 3.2. Deposition of mAbs in the Egg White of KI Chickens

**Figure 2:**
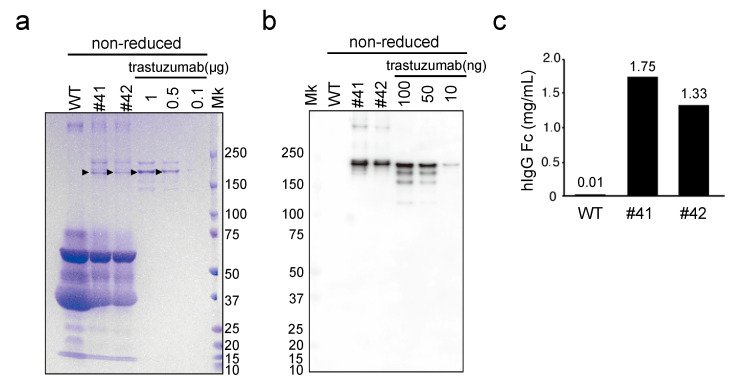
Evaluation of mAbs deposited in KI egg whites using SDS-PAGE and immunoblotting, under nonreducing conditions. (**a**) SDS-PAGE of WT and mAb KI (#41 and #42) egg whites, and recombinant trastuzumab, stained with Coomassie Brilliant Blue. The egg white samples (20-fold diluted) and trastuzumab (0.1–1 µg) were loaded as indicated at the top of the lanes. Arrowheads indicate the major bands estimated to be fully assembled mAbs. Mk: protein standard marker. (**b**) Immunoblotting with the anti-human immunoglobulin G (hIgG) Fc antibody against WT, mAb KI egg whites, and recombinant trastuzumab. The egg white samples (200-fold diluted) and trastuzumab (10–100 ng) were loaded as indicated. (**c**) Concentration of hIgG estimated using immunoblotting. Each immunoreactive signal was quantified using ImageJ, and the relative concentrations of hIgG in egg whites were estimated using trastuzumab as a standard.

After sexual maturation, the KI hens began laying eggs (hereafter, denoted as KI eggs). The average number of KI eggs laid in the first 100 days was 78.7 ± 4.0 (n = 3). As KI eggs were obtained, we attempted to detect whether the white of KI eggs (denoted as KI egg white) contained the mAb. The egg whites from two KI female chickens (#41 and #42), a WT chicken, as well as the commercial anti-HER2 antibody (trastuzumab) were subjected to SDS-PAGE analysis under nonreducing conditions, followed by CBB staining (Figure 2a). CBB-stained protein bands with mass >150 kDa were observed in the KI egg white sample, but not in the WT egg white sample. Notably, stained protein bands of similar molecular mass were also observed in the trastuzumab sample lanes, suggesting that intact assembled mAbs were present in the KI egg white. To confirm the presence of humanized mAbs in KI egg whites, we performed immunoblotting with an antibody against hIgG Fc. Consistent with the CBB staining, a major signal was detected, similar in size to trastuzumab, in KI egg whites, but not in the WT egg white (Figure 2b). Therefore, the presence of humanized mAbs in the KI egg whites was confirmed. The concentrations of mAbs in KI egg whites were estimated to be 1.75 and 1.33 mg/mL for egg #41 and #42, respectively, by comparison with the protein band intensities of trastuzumab presented in Figure 2b (Figure 2c).

**Figure 3:**
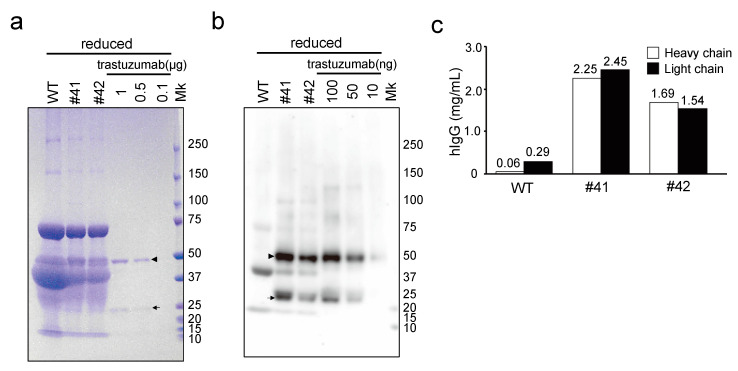
Evaluation of the mAbs deposited in KI egg whites using SDS-PAGE and immunoblotting, under reducing conditions. (**a**) SDS-PAGE of WT egg whites, mAb KI (#41 and #42) egg whites, and recombinant mAb trastuzumab, stained with Coomassie Brilliant Blue. The egg white samples (20-fold diluted) and trastuzumab (0.1–1 µg) were loaded as indicated at the top of the lanes. The arrowhead and arrow indicate the position of the heavy and light chains of hIgG, respectively. Mk: protein standard marker. (**b**) Immunoblotting with the anti-hIgG (H + L) antibody against WT, mAb KI egg whites, and recombinant trastuzumab. The arrowhead and arrow indicate the position of the heavy and light chains of hIgG, respectively. (**c**) The concentration of hIgG as estimated by immunoblotting with trastuzumab as the standard. Immunoreactive signals corresponding to the heavy and light chains were quantified using ImageJ and are represented using white and black bars, respectively.

The mAbs in KI egg whites were further analyzed by CBB staining and immunoblotting under reducing conditions. Although the individual heavy and light chains of mAbs in the KI egg whites were not definitively identified with CBB staining, because of overlap with other egg white proteins (Figure 3a), they were detected by immunoblotting using anti-hIgG (H + L) antibodies and were found to have motilities consistent with those of trastuzumab heavy and light chains (Figure 3b). The concentrations of mAbs in KI egg whites were 2.25 and 2.45 mg/mL for egg #41, as calculated by comparing the intensities of heavy and light chain bands, respectively, with those of trastuzumab (Figure 3c); similarly, the concentrations in egg #42 were estimated as 1.69 and 1.54 mg/mL, respectively. These results indicate that full-length heavy and light chains were present in KI egg whites in almost equal amounts and as independent subunits. Taken together with the results in Figure 2a,b, it is suggested that the majority of mAbs in KI egg whites consisted of two heavy chains and two light chains.

### 3.3. Quantitation of mAbs in the Egg Whites of KI Chickens

**Figure 4:**
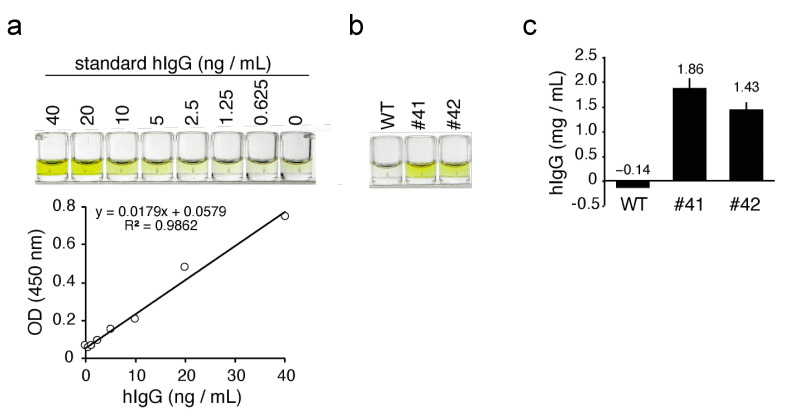
ELISA for hIgG in KI egg whites. (**a**) Top: color development of standard hIgG in ELISA. The concentrations of the standard hIgG samples are shown at the top of the wells. Bottom: standard curve based on the optical density (OD) values. *R^2^* is the linear regression *R*-squared value. (**b**) Color development of hIgG from the WT and KI egg whites in ELISA. The WT and KI egg whites were diluted 100,000-fold and subjected to ELISA. The OD value of samples from WT, #41, and #42 eggs were 0.04, 0.42, and 0.33, respectively. (**c**) The concentration of hIgG in egg whites as estimated by ELISA. Bars show the mean ± standard deviation of the concentration of hIgG in each egg white sample (n = 3).

To quantify the concentration of mAbs in KI egg whites more precisely, we performed a sandwich ELISA for the detection of hIgG. Standard hIgG showed linear color development in the concentration range from 0.625 ng/mL to 40 ng/mL, with a linear regression *R*-squared value of 0.986 (Figure 4a). In the same ELISA experiment, diluted KI egg whites, but not the WT egg whites, exhibited a similar color development (Figure 4b). The average concentration of mAbs in egg #41 and 42 was estimated as 1.86 ± 0.22 (n = 3) and 1.43 ± 0.16 (n = 3) mg/mL, respectively, with reference to the hIgG standard (Figure 4c). 

**Figure 5:**
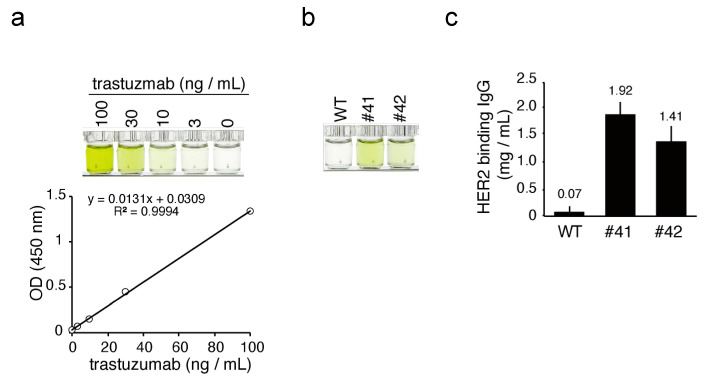
ELISA for HER2 binding hIgG in KI egg whites. (**a**) Top: color development of standard trastuzumab in ELISA. The concentrations of the standard trastuzumab samples are shown at the top of the wells. Bottom: standard curve based on the optical density (OD) values. *R^2^* is the linear regression *R*-squared value. (**b**) Color development of HER2 binding hIgG from WT and KI egg whites in ELISA. The WT and KI egg whites were diluted 100,000-fold and subjected to ELISA. The OD values of samples from WT, #41, and #42 eggs were 0.03, 0.34, and 0.17, respectively. (**c**) The concentrations of HER2 binding hIgG in egg whites as estimated by ELISA. Bars show the mean ± standard deviation of the concentration of HER2 binding hIgG in each egg white sample (*n* = 3).

Next, we addressed whether the mAbs in KI egg whites had antigen binding activity. Because KI hens were designed to deposit humanized anti-HER2 antibody into the egg whites, we analyzed the binding activity against recombinant HER2 protein. The KI and WT egg whites, as well as trastuzumab, were incubated in a microtiter plate coated with recombinant HER2, and their binding affinities were analyzed by ELISA (trastuzumab ELISA). Standard trastuzumab exhibited a dose-dependent color development within the 3–100 ng/mL range, with a linear regression *R*-squared value of 0.999 (Figure 5a). In the same ELISA, diluted egg white samples from KI egg whites #41 and #42, but not from the WT sample, developed color (Figure 5b), suggesting that the mAbs in the KI egg white could recognize recombinant HER2 as an antigen. The average concentration of anti-HER2 mAbs in the whites of KI eggs #41 and #42 was estimated to be 1.92 ± 0.23 (n = 3) and 1.41 ± 0.29 (n = 3) mg/mL, respectively, using trastuzumab as a known reference (Figure 5c). Because the concentrations of mAbs in KI egg whites estimated by hIgG ELISA (Figure 4c) and trastuzumab ELISA (Figure 5c) were almost the same, it is suggested that the antigen binding affinity of mAbs in KI egg whites is close to that of trastuzumab.

### 3.4. Antigen-Specific Binding of mAbs in the Egg Whites of KI Chickens

**Figure 6:**
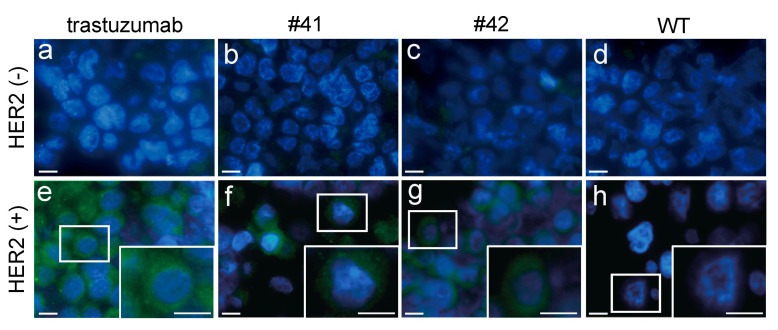
HER2-specific binding of the mAbs deposited in KI egg whites. Sections of HER2-negative (HER2(−), (**a**–**d**) and positive (HER2(+), (**e**–**h**) cells were treated with trastuzumab (**a**,**e**), KI egg whites from chicken #41 (**b**,**f**) and chicken #42 (**c**,**g**) and WT egg whites (**d**,**h**), subsequently examined using immunofluorescence. The expression of HER2 was visualized using an Alexa Fluor 488-conjugated tertiary antibody (Green). The cell nuclei were counterstained with DAPI. Enlarged insets of HER2-positive cells are shown in (**e**–**h**). The scale bar represents 10 µm.

Lastly, we examined whether the mAbs deposited in KI egg whites could specifically bind to the HER2 antigen expressed on cells. For this purpose, we performed immunofluorescence analysis against HER2-positive and -negative cells. The concentrations of trastuzumab and egg-derived mAbs (estimated as hIgG, Figure 4c) were adjusted to 1 µg/mL and used as primary antibodies. The binding of primary antibodies to the cells was detected by mouse anti-hIgG and visualized with Alexa Fluor 488-conjugated anti-mouse IgG (Figure 6). The binding of trastuzumab and KI egg-derived mAbs was apparent in HER2-positive cells (Figure 6e–g), but not in HER2-negative cells (Figure 6a–c). Fluorescence signals associated with binding of antibodies isolated from WT egg whites were not detected in HER2-positive or -negative cells (Figure 6d,h). Careful analysis of the images obtained for cells treated with either trastuzumab or mAbs obtained from KI egg white indicated that the localization of fluorescence signals was similarly predominantly detected within the cytosol and not in the nuclei (insets of Figure 6e–g). The specific signal localization observed is consistent with the expression pattern of HER2 in positive tumor cells [20]. Taken together, these results indicate that mAbs in KI egg whites specifically bind to HER2 in cells and that binding specificity and affinity are comparable to those of commercial trastuzumab.

## 4. Discussion

In this study, we efficiently produced fully assembled anti-HER2 mAbs in egg whites by integrating the mAb gene at the initiation site of the *OVA* locus. We further demonstrated that anti-HER2 mAbs deposited in the KI egg white showed similar HER2-binding properties in comparison to the commercially available therapeutic antibody, trastuzumab.

The concentration of mAbs deposited in the egg whites of KI chickens was in the 1.4–1.9 mg/mL range (Figure 4c), which is 80- to 1000-fold higher than the previously reported mAb expression levels in similar transgenic chickens employing 3.5 kb of the *OVA* promoter, to drive expression, with random gene integration [13]. Similarly, in our previous study, the concentration of human IFN-β deposited in the egg whites of KI chickens was 1.9–4.4 mg/mL [18], which is 15- to 1200-fold higher than other reports using transgenic chickens for human IFN-β production with 2.8 kb of the *OVA* promoter, to drive expression and random integration of *IFN-β* [16]. In both our present and our previous KI chicken studies, the foreign genes were integrated into the initiation site of *OVA*. This approach resulted in abundant levels of foreign proteins deposited into the KI egg whites, in comparison to transgenic systems employing random gene integration. Therefore, it is conceivable that transgene insertion at the *OVA* locus is a reliable method to establish an efficient transgenic chicken bioreactor.

The antigen binding of anti-HER2 mAbs was analyzed directly using diluted KI egg whites, without purification (Figure 5c). Nevertheless, the anti-HER2 mAbs exhibited almost equal antigen binding affinity to that of trastuzumab, a therapeutic anti-HER2 antibody. This result indicates that fully assembled mAbs were efficiently synthesized in KI chicken magnum, and stably deposited into the egg whites. Thus, it is anticipated that the chicken bioreactor system developed in this study is well suited for mAb production and has great potential as an alternative method for commercial manufacturing. In addition, the development of a more cost-effective approach to mAb production is highly desirable. Although production costs are dependent on many variables, including production scale and methods, we estimate the cost of 1 g of mAb to be >40 USD, using both cell culture and plant bioreactor systems [21,22,23]. According to our current study, a KI egg with 30 mL of egg white would contain 42–57 mg of mAbs. Because commercial egg production costs are approximately $1 per dozen eggs [24], 1 g of mAbs could be produced for 1.5–2.0 USD using our chicken bioreactor system. It is important to note that this is a rough estimate, as it does not consider several parameters, including costs associated with the development of KI chickens, their genotyping, and the egg-laying period of KI hens. However, these factors are limited, and we believe that our chicken bioreactor system represents a cost-effective upstream process for therapeutic mAb production. For commercial manufacturing of therapeutic mAbs, the costs incurred in the downstream process (i.e., mAb purification) and approvals (or drug certification) by regulatory bodies should also be considered. However, the cost advantage of the chicken bioreactor system vis-à-vis the other mAb production systems has not been evaluated. It was surmised that the costs for downstream processing of mAbs produced using the chicken bioreactor should be equivalent to those for mAbs produced using the mammalian cell culture methods [17]. Although the costs for regulatory approvals are not in the public domain, recently, a therapeutic recombinant protein produced using a chicken bioreactor was approved by the FDA, suggesting that these processes are not unrealistically expensive [25].

It is estimated that the typical production scale of commercial therapeutic mAbs is on the order of hundreds of kg/year or less [26]. To meet this demand using the chicken bioreactor system, tens of thousands of KI hens will be required annually. This is feasible considering the high fertility of chickens. Furthermore, it is expected that one transgenic rooster could be used to breed over a hundred thousand transgenic offspring in a single year [11]. In addition, a chicken flock is easily scalable; thus, the system can be adapted to suit variable levels of production demands. Therefore, the chicken bioreactor system is a suitable approach for the manufacture of commercial therapeutic mAbs.

In conclusion, a chicken bioreactor system, based on mAb gene KI at the *OVA* locus, can efficiently produce mAbs with adequate antigen binding capacity and has the potential to be a viable alternative production system for commercial mAbs. Furthermore, the approach is cost-effective and easily scalable. Although further studies are required to establish downstream processes that will be required to enable medical applications, the production of therapeutic mAbs using KI chicken bioreactors is a promising cost-effective technology for the production of therapeutic mAbs.

## Data Availability

The data presented in this study are available from the corresponding author on reasonable request.

## Supplementary Materials

The following are available online at https://www.mdpi.com/2073-4425/12/1/38/s1, Figure S1: DNA sequence of mAb donor construct and location of features.

## References

[1] Lu R.M., Hwang Y.C., Liu I.J., Lee C.C., Tsai H.Z., Li H.J., Wu H.C. (2020). Development of Therapeutic Antibodies for the Treatment of Diseases. J. Biomed. Sci..

[2] Shukla A.A., Wolfe L.S., Mostafa S.S., Norman C. (2017). Evolving Trends in mAb Production Processes. Bioeng. Transl. Med..

[3] Ecker D.M., Jones S.D., Levine H.L. (2015). The Therapeutic Monoclonal Antibody Market. mAbs.

[4] Singh S., Kumar N.K., Dwiwedi P., Charan J., Kaur R., Sidhu P., Chugh V.K. (2018). Monoclonal Antibodies: A Review. Curr. Clin. Pharmacol..

[5] Valente K.N., Levy N.E., Lee K.H., Lenhoff A.M. (2018). Applications of Proteomic Methods for CHO Host Cell Protein Characterization in Biopharmaceutical Manufacturing. Curr. Opin. Biotechnol..

[6] Maksimenko O.G., Deykin A.V., Khodarovich Y.M., Georgiev P.G. (2013). Use of Transgenic Animals in Biotechnology: Prospects and Problems. Acta Nat..

[7] Tada M., Tatematsu K., Ishii-Watabe A., Harazono A., Takakura D., Hashii N., Sezutsu H., Kawasaki N. (2015). Characterization of Anti-CD20 Monoclonal Antibody Produced by Transgenic Silkworms (Bombyx mori). mAbs.

[8] Bertolini L.R., Meade H., Lazzarotto C.R., Martins L.T., Tavares K.C., Bertolini M., Murray J.D. (2016). The Transgenic Animal Platform for Biopharmaceutical Production. Transgen. Res..

[9] Dyck M.K., Lacroix D., Pothier F., Sirard M.A. (2003). Making Recombinant Proteins in Animals-Different Systems, Different Applications. Trends Biotechnol..

[10] Bahrami S., Amiri-Yekta A., Daneshipour A., Jazayeri S.H., Mozdziak P.E., Sanati M.H., Gourabi H. (2020). Designing A Transgenic Chicken: Applying New Approaches toward A Promising Bioreactor. Cell J..

[11] Lillico S.G., McGrew M.J., Sherman A., Sang H.M. (2005). Transgenic Chickens as Bioreactors for Protein-Based Drugs. Drug Discov. Today.

[12] Zhu L., van de Lavoir M.C., Albanese J., Beenhouwer D.O., Cardarelli P.M., Cuison S., Deng D.F., Deshpande S., Diamond J.H., Green L. (2005). Production of Human Monoclonal Antibody in Eggs of Chimeric Chickens. Nat. Biotechnol..

[13] Kim Y.M., Park J.S., Kim S.K., Jung K.M., Hwang Y.S., Han M., Lee H.J., Seo H.W., Suh J.Y., Han B.K. (2018). The Transgenic Chicken Derived Anti-CD20 Monoclonal Antibodies Exhibits Greater Anti-Cancer Therapeutic Potential with Enhanced Fc Effector Functions. Biomaterials.

[14] Liu T.X., Wu H.Y., Cao D.N., Li Q.Y., Zhang Y.Q., Li N., Hu X.X. (2015). Oviduct-Specific Expression of Human Neutrophil Defensin 4 in Lentivirally Generated Transgenic Chickens. PLoS ONE.

[15] Nishijima K., Iijima S. (2013). Transgenic Chickens. Dev. Growth Differ..

[16] Lillico S.G., Sherman A., McGrew M.J., Robertson C.D., Smith J., Haslam C., Barnard P., Radcliffe P.A., Mitrophanous K.A., Elliot E.A. (2007). Oviduct-Specific Expression of Two Therapeutic Proteins in Transgenic Hens. Proc. Natl. Acad. Sci. USA.

[17] Herron L.R., Pridans C., Turnbull M.L., Smith N., Lillico S., Sherman A., Gilhooley H.J., Wear M., Kurian D., Papadakos G. (2018). A Chicken Bioreactor for Efficient Production of Functional Cytokines. BMC Biotechnol..

[18] Oishi I., Yoshii K., Miyahara D., Tagami T. (2018). Efficient Production of Human Interferon beta in the White of Eggs from Ovalbumin Gene-Targeted Hens. Sci. Rep..

[19] Oishi I., Yoshii K., Miyahara D., Kagami H., Tagami T. (2016). Targeted Mutagenesis in Chicken Using CRISPR/Cas9 System. Sci. Rep..

[20] Pereira P.M.R., Sharma S.K., Carter L.M., Edwards K.J., Pourat J., Ragupathi A., Janjigian Y.Y., Durack J.C., Lewis J.S. (2018). Caveolin-1 Mediates Cellular Distribution of HER2 and Affects Trastuzumab Binding and Therapeutic Efficacy. Nat. Commun..

[21] Kelley B. (2009). Industrialization of mAb Production Technology: The Bioprocessing Industry at a Crossroads. mAbs.

[22] Klutz S., Holtmann L., Lobedann M., Schembecker G. (2016). Cost Evaluation of Antibody Production Processes in Different Operation Modes. Chem. Eng. Sci..

[23] Nandi S., Kwong A.T., Holtz B.R., Erwin R.L., Marcel S., McDonald K.A. (2016). Techno-Economic Analysis of a Transient Plant-Based Platform for Monoclonal Antibody Production. mAbs.

[24] Matthews W.A., Sumner D.A. (2015). Effects of Housing System on the Costs of Commercial Egg Production. Poult. Sci..

[25] Sheridan C.F.D.A. (2016). FDA Approves ‘Farmaceutical’ Drug from Transgenic Chickens. Nat. Biotechnol..

[26] Decker J.S., Menacho-Melgar R., Lynch M.D. (2020). Low-Cost, Large-Scale Production of the Anti-Viral Lectin Griffithsin. Front. Bioeng. Biotechnol..

