# Supplementary material for: Production of Recombinant Monoclonal Antibodies in the Egg White of Gene-Targeted Transgenic Chickens
Source: Genes (Basel). 2020 Dec 30;12(1):38. doi: 10.3390/genes12010038 (PMC7823952; doi:10.3390/genes12010038)
Supplement: Supplementary file 1 [file genes-12-00038-s001.pdf]

Figure S1. DNA sequence of mAb donor construct and location of features.

OVA 5′ homology region: 1–2791

Lysozyme signal: 2792–2845

Heavy chain of the anti-HER2 mAb: 2846–4194

Furin 2A peptide: 4195–4278

Lysozyme signal: 4279–4332

Light chain of the anti-HER2 mAb: 4333–4977

bGH polyadenylation signal: 4982–5199

Neomycin resistance gene unit: 5206–6896

OVA 3′ homology region: 6908–10102

```
   1 GTCGACCTTA AGTCCTCAGA CTTGGCAAGG AGAATGTAGA TTTCTACAGT ATATATGTTT
  61 TCACAAAAGG AAGGAGAGAA ACAAAAGAAA ATGGCACTGA CTAAACTTCA GCTAGTGGTA
 121 TAGGAAAGTA ATTCTGCTTA ACAGAGATTG CAGTGATCTC TATGTATGTC CTGAAGAATT
 181 ATGTTGTACT TTTTTCCCTC ATTTTTAAAT CAAACAGTGC TTTACAGAGG TCAGAATGGT
 241 TTCTTTACTG TTTGTCAATT CTATTATTTC AATACAGAAC AATAGCTTCT ATAACTGAAA
 301 TATATTTGCT ATTGTATATT ATGATTGTCC CTCGAACCAT GAACACTCCT CCAGCTGAAT
 361 TTCACAATTC CTCTGTCATC TGCCAGGCCA TTAAGTTATT CATGGAAGAT CTTTGAGGAA
 421 CACTGCAAGT TCATATCATA AACACATTTG AAATTGAGTA TTGTTTTGCA TTGTATGGAG
 481 CTATGTTTTG CTGTATCCTC AGAAAAAAAG TTTGTTATAA AGCATTCACA CCCATAAAAA
 541 GATAGATTTA AATATTCCAG CTATAGGAAA GAAAGTGCGT CTGCTCTTCA CTCTAGTCTC
 601 AGTTGGCTCC TTCACATGCA CGCTTCTTTA TTTCTCCTAT TTTGTCAAGA AAATAATAGG
 661 TCACGTCTTG TTCTCACTTA TGTCCTGCCT AGCATGGCTC AGATGCACGT TGTACATACA
 721 AGAAGGATCA AATGAAACAG ACTTCTGGTC TGTTACTACA ACCATAGTAA TAAGCACACT
 781 AACTAATAAT TGCTAATTAT GTTTTCCATC TCCAAGGTTC CCACATTTTT CTGTTTTCTT
 841 AAAGATCCCA TTATCTGGTT GTAACTGAAG CTCAATGGAA CATGAGCAAT ATTTCCCAGT
 901 CTTCTCTCCC ATCCAACAGT CCTGATGGAT TAGCAGAACA GGCAGAAAAC ACATTGTTAC
 961 CCAGAATTAA AAACTAATAT TTGCTCTCCA TTCAATCCAA AATGGACCTA TTGAAACTAA
1021 AATCTAACCC AATCCCATTA AATGATTTCT ATGGCGTCAA AGGTCAAACT TCTGAAGGGA
1081 ACCTGTGGGT GGGTCACAAT TCAGGCTATA TATTCCCCAG GGCTCAGCCA GTGTCTGTAC
1141 ATACAGCTAG AAAGCTGTAT TGCCTTTAGC ACTCAAGCTC AAAAGGTAAG CAACTCTCTG
1201 GAATTACCTT CTCTCTATAT TAGCTCTTAC TTGCACCTAA ACTTTAAAAA ATTAACAATT
1261 ATTGTGTTAT GTGTTGTATC TTTAAGGGTG AAGTACCTGC GTGATACCCC CTATAAAAAC
1321 TTCTCACCTG TGTATGCATT CTGCACTATT TTATTATGTG TAAAAGCTTT GTGTTTGTTT
1381 TCAGGAGGCT TATTCTTTGT GCTTAAAATA TGTTTTTAAT TTCAGAACAT CTTATCCTGT
1441 CGTTCACTAT CTGATATGCT TTGCAGTTTG CCTGATTAAC TTCTAGCCCT ACAGAGTGCA
1501 CAGAGAGCAA AATCATGGTG TTCAGTGAAT TCTGGGGAGT TATTTTAATG TGAAAATTCT
1561 CTAGAAGTTT AATTCCTGCA AAGTGCAGCT GCTGATCACT ACACAAGATA AAAATGTGGG
1621 GGGTGCATAA ACGTATATTC TTACAATAAT AGATACATGT GAACTTGTAT ACAGAAAAGA
1681 AAATGAGAAA AATGTGTGTG CGTATACTCA CACACGTGGT CAGTAAAAAC TTTTGAGGGG
1741 TTTAATACAG AAAATCCAAT CCTGAGGCCC CAGCACTCAG TACGCATATA AAGGGCTGGG
1801 CTCTGAAGGA CTTCTGACTT TCACAGATTA TATAAATCTC AGGAAAGCAA CTAGATTCAT
1861 GCTGGCTCCA AAAGCTGTGC TTTATATAAG CACACTGGCT ATACAATAGT TGTACAGTTC
1921 AGCTCTTTAT AATAGAAACA GACAGAACAA GTATAAATCT TCTATTGGTC TATGTCATGA
1981 ACAAGAATTC ATTCAGTGGC TCTGTTTTAT AGTAAACATT GCTATTTTAT CATGTCTGCA
2041 TTTCTCTTCT GTCTGAATGT CACCACTAAA ATTTAACTCC ACAGAAAGTT TATACTACAG
2101 TACACATGCA TATCTTTGAG CAAAGCAAAC CATACCTGAA AGTGCAATAG AGCAGAATAT
2161 GAATTACATG CGTGTCTTTC TCCTAGACTA CATGACCCCA TATAAATTAC ATTCCTTATC
2221 TATTCTGCCA TCACCAAAAC AAAGGTAAAA ATACTTTTGA AGATCTACTC ATAGCAAGTA
2281 GTGTGCAACA AACAGATATT TCTCTACATT TATTTTTAGG GAATAAAAAT AAGAAATAAA
2341 ATAGTCAGCA AGCCTCTGCT TTCTCATATA TCTGTCCAAA CCTAAAGTTT ACTGAAATTT
2401 GCTCTTTGAA TTTCCAGTTT TGCAAGCCTA TCAGATTGTG TTTTAATCAG AGGTACTGAA
2461 AAGTATCAAT GAATTCTAGC TTTCACTGAA CAAAAATATG TAGAGGCAAC TGGCTTCTGG
2521 GACAGTTTGC TACCCAAAAG ACAACTGAAT GCAAATACAT AAATAGATTT ATGAATATGG
```

2581 TTTTGAACAT GCACATGAGA GGTGGATATA GCAACAGACA CATTACCACA GAATTACTTT
2641 AAAACTACTT GTTAACATTT AATTGCCTAA AAACTGCTCG TAATTTACTG TTGTAGCCTA
2701 CCATAGAGTA CCCTGCATGG TACTATGTAC AGCATTCCAT CCTTACATTT TCACTGTTCT
2761 GCTGTTTGCT CTAGACAACT CAGAGTTCAC CATGAGGTCT TTGCTAATCT TGGTGCTTTG
2821 CTTCCTGCCC CTGGCTGCTC TGGGGGAGGT CCAGCTGGTG GAGTCCGGCG
GAGGGCTGGT
2881 CCAGCCTGGA GGCAGCCTGA GACTGAGCTG TGCTGCCAGC GGGTTCAATA TCAAGGATAC
2941 CTACATCCAC TGGGTGAGGC AGGCCCCCGG AAAGGGCCTG GAATGGGTGG
CCAGGATTTA
3001 CCCAACTAAT GGGTATACTC GGTACGCCGA TTCCGTCAAA GGCAGATTTA CCATTAGCGC
3061 AGACACCAGC AAAAACACAG CATACCTGCA GATGAACTCC CTGAGAGCTG AAGACACAGC
3121 TGTGTATTAT TGCTCCCGGT GGGGGGGCGA CGGCTTTTAT GCCATGGACT ACTGGGGCCA
3181 GGGAACCCTG GTCACCGTCT CCTCAGCCTC CACCAAGGGC CCATCGGTCT
TCCCCCTGGC
3241 ACCCTCCTCC AAGAGCACCT CTGGGGGCAC AGCAGCCCTG GGCTGCCTGG
TCAAGGACTA
3301 CTTCCCCGAA CCGGTGACGG TGTCGTGGAA CTCAGGCGCC CTGACCAGCG
GCGTGCACAC
3361 CTTCCCGGCT GTCCTACAGT CCTCAGGACT CTACTCCCTC AGCAGCGTGG TGACCGTGCC
3421 CTCCAGCAGC TTGGGCACCC AGACCTACAT CTGCAACGTG AATCACAAGC CCAGCAACAC
3481 CAAGGTGGAC AAGAAAGTTG AGCCCAAATC TTGTGACAAA ACTCACACAT GCCCACCGTG
3541 CCCAGCACCT GAACTCCTGG GGGGACCGTC AGTCTTCCTC TTCCCCCCAA AACCCAAGGA
3601 CACCCTCATG ATCTCCCGGA CCCCTGAGGT CACATGCGTG GTGGTGGACG
TGAGCCACGA
3661 AGACCCTGAG GTCAAGTTCA ACTGGTACGT GGACGGCGTG GAGGTGCATA ATGCCAAGAC
3721 AAAGCCGCGG GAGGAGCAGT ACAACAGCAC GTACCGTGTG GTCAGCGTCC
TCACCGTCCT
3781 GCACCAGGAC TGGCTGAATG GCAAGGAGTA CAAGTGCAAG GTCTCCAACA AAGCCCTCCC
3841 AGCCCCCATC GAGAAAACCA TCTCCAAAGC CAAAGGGCAG CCCCGAGAAC CACAGGTGTA
3901 CACCCTGCCC CCATCCCGGG ATGAGCTGAC CAAGAACCAG GTCAGCCTGA
CCTGCCTGGT
3961 CAAAGGCTTC TATCCCAGCG ACATCGCCGT GGAGTGGGAG AGCAATGGGC
AGCCGGAGAA
4021 CAACTACAAG ACCACGCCTC CCGTGCTGGA CTCCGACGGC TCCTTCTTCC TCTACAGCAA
4081 GCTCACCGTG GACAAGAGCA GGTGGCAGCA GGGGAACGTC TTCTCATGCT
CCGTGATGCA
4141 TGAGGCTCTG CACAACCACT ACACGCAGAA GAGCCTCTCC CTGTCTCCGG GTAAACGGGC
4201 CAAGAGAGCC CCCGTGAAGC AGACCCTGAA CTTCGACCTG CTGAAGCTGG
CCGGCGACGT
4261 GGAGTCCAAC CCCGGCCCCA TGCGCTCCCT CTTGATTCTC GTCTTGTGTT TTTTGCCACT
4321 GGCCGCTCTC GGCGACATAC AGATGACCCA GAGCCCCTCT TCTCTCTCAG CATCAGTCGG
4381 CGACAGGGTC ACAATTACCT GCCGGGCTAG CCAAGATGTG AACACAGCCG TGGCTTGGTA
4441 TCAGCAGAAA CCTGGGAAGG CCCCAAAACT GCTGATTTAT TCTGCTAGCT TCCTGTATTC
4501 TGGGGTGCCT TCCAGATTCT CCGGATCCAG ATCCGGCACT GATTTCACAC TGACCATCAG
4561 CAGCCTCCAG CCCGAGGATT TTGCAACATA CTACTGTCAG CAACACTACA CTACTCCTCC
4621 AACCTTTGGC CAAGGCACCA AGGTTGAAAT CAAGCGTACG GTGGCTGCAC CATCTGTCTT
4681 CATCTTCCCG CCATCTGATG AGCAGTTGAA ATCTGGAACT GCCTCTGTTG TGTGCCTGCT
4741 GAATAACTTC TATCCCAGAG AGGCCAAAGT ACAGTGGAAG GTGGATAACG CCCTCCAATC
4801 GGGTAACTCC CAGGAGAGTG TCACAGAGCA GGACAGCAAG GACAGCACCT
ACAGCCTCAG
4861 CAGCACCCTG ACGCTGAGCA AAGCAGACTA CGAGAAACAC AAAGTCTACG CCTGCGAAGT
4921 CACCCATCAG GGCCTGAGCT CGCCCGTCAC AAAGAGCTTC AACAGGGGAG AGTGTTAGTC
4981 GACTCGCTGA TCAGCCTCGA CTTTGCCTTC TAGTTGCCAG CCATCTGTTG TTTGCCCCTC
5041 CCCCGTGCCT TCCTTGACCC TGGAAGGTGC CACTCCCACT GTCCTTTCCT AATAAAATGA
5101 GGAAATTGCA TCGCATTGTC TGAGTAGGTG TCATTCTATT CTGGGGGGTG GGGTGGGGCA
5161 GGACAGCAAG GGGGAGGATT GGGAAGACAA TAGCAGGCAG TCGAGTTCAA ATATGTATCC
5221 GCTCATGAGA CAATAACCCT GATAAATGCT TCAATAATAT TGAAAAAGGA AGAGTCCTGA
5281 GGCGGAAAGA ACCAGCTGTG GAATGTGTGT CAGTTAGGGT GTGGAAAGTC
CCCAGGCTCC
5341 CCAGCAGGCA GAAGTATGCA AAGCATGCAT CTCAATTAGT CAGCAACCAG GTGTGGAAAG
5401 TCCCCAGGCT CCCCAGCAGG CAGAAGTATG CAAAGCATGC ATCTCAATTA GTCAGCAACC
5461 ATAGTCCCGC CCCTAACTCC GCCCATCCCG CCCCTAACTC CGCCCAGTTC CGCCCATTCT
5521 CCGCCCCATG GCTGACTAAT TTTTTTTATT TATGCAGAGG CCGAGGCCGC CTCGGCCTCT

5581 GAGCTATTCC AGAAGTAGTG AGGAGGCTTT TTTGGAGGCC TAGGCTTTTG CAAAGATCGA
5641 TCAAGAGACA GGATGAGGAT CGTTTCGCAT GATTGAACAA GATGGATTGC ACGCAGGTTC
5701 TCCGGCCGCT TGGGTGGAGA GGCTATTCGG CTATGACTGG GCACAACAGA
CAATCGGCTG
5761 CTCTGATGCC GCCGTGTTCC GGCTGTCAGC GCAGGGGCGC CCGGTTCTTT TTGTCAAGAC
5821 CGACCTGTCC GGTGCCCTGA ATGAACTGCA AGACGAGGCA GCGCGGCTAT
CGTGGCTGGC
5881 CACGACGGGC GTTCCTTGCG CAGCTGTGCT CGACGTTGTC ACTGAAGCGG
GAAGGGACTG
5941 GCTGCTATTG GGCGAAGTGC CGGGGCAGGA TCTCCTGTCA TCTCACCTTG CTCCTGCCGA
6001 GAAAGTATCC ATCATGGCTG ATGCAATGCG GCGGCTGCAT ACGCTTGATC CGGCTACCTG
6061 CCCATTCGAC CACCAAGCGA AACATCGCAT CGAGCGAGCA CGTACTCGGA
TGGAAGCCGG
6121 TCTTGTCGAT CAGGATGATC TGGACGAAGA GCATCAGGGG CTCGCGCCAG CCGAACTGTT
6181 CGCCAGGCTC AAGGCGAGCA TGCCCGACGG CGAGGATCTC GTCGTGACCC
ATGGCGATGC
6241 CTGCTTGCCG AATATCATGG TGGAAAATGG CCGCTTTTCT GGATTCATCG ACTGTGGCCG
6301 GCTGGGTGTG GCGGACCGCT ATCAGGACAT AGCGTTGGCT ACCCGTGATA TTGCTGAAGA
6361 GCTTGGCGGC GAATGGGCTG ACCGCTTCCT CGTGCTTTAC GGTATCGCCG CTCCCGATTC
6421 GCAGCGCATC GCCTTCTATC GCCTTCTTGA CGAGTTCTTC TGAGCGGGAC TCTGGGGTTC
6481 GAAATGACCG ACCAAGCGAC GCCCAACCTG CCATCACGAG ATTTCGATTC CACCGCCGCC
6541 TTCTATGAAA GGTTGGGCTT CGGAATCGTT TTCCGGGACG CCGGCTGGAT GATCCTCCAG
6601 CGCGGGGATC TCATGCTGGA GTTCTTCGCC CACCCTAGGG GGAGGCTAAC
TGAAACACGG
6661 AAGGAGACAA TACCGGAAGG AACCCGCGCT ATGACGGCAA TAAAAAGACA GAATAAAACG
6721 CACGGTGTTG GGTCGTTTGT TCATAAACGC GGGGTTCGGT CCCAGGGCTG GCACTCTGTC
6781 GATACCCCAC CGAGACCCCA TTGGGGCCAA TACGCCCGCG TTTCTTCCTT TTCCCCACCC
6841 CACCCCCCAA GTTCGGGTGA AGGCCCAGGG CTCGCAGCCA ACGTCGGGGC
GGCAGGTACC
6901 AGTGATAATC GAATTCCCGC GGCCGCGGGA ATTCGATTCC ATCGGTGCAG CAAGCATGGA
6961 ATTTTGTTTT GATGTATTCA AGGAGCTCAA AGTCCACCAT GCCAATGAGA ACATCTTCTA
7021 CTGCCCCATT GCCATCATGT CAGCTCTAGC CATGGTATAC CTGGGTGCAA AAGACAGCAC
7081 CAGGACACAG ATAAATAAGG TGAGCCTACA GTTAAAGATT AAAACCTTTG CCCTGCTCAA
7141 TGGAGCCACA GCACTTAATT GTATGATAAT GTCCCTTGGA AACTGCATAG CTCAGAGGCT
7201 GAAAATCTGA AACCAGAGTT ATCTAAAAGT GTGGCCACCT CCAACTCCCA GAGTGTTACC
7261 CAAATGCACT AGCTAGAAAT CTTGAAACTG GATTGCATAA CTTCTTTTTG TCATAACCAT
7321 TATTTCAGCT ACTATTATTT TCAATTACAG GTTGTTCGCT TTGATAAACT TCCAGGATTC
7381 GGAGACAGTA TTGAAGCTCA GGTACAGAAA TAATTTCACC TCCTTCTCTA TGTCCCTTTC
7441 CTCTGGAAGC AAAATACAGC AGATGAAGCA ATCTCTTAGC TGTTCCAAGC CCTCTCTGAT
7501 GAGCAGCTAG TGCTCTGCAT CCAGCAGTTG GGAGAACACT GTTCATAAGA ACAGAGAAAA
7561 AGAAGGAAGT AACAGGGGAT TCAGAACAAA CAGAAGATAA AACTCAGGAC AAAAATACCG
7621 TGTGAATGAG GAAACTTGTG GATATTTGTA CGCTTAAGCA AGACAGCTAG ATGATTCTGG
7681 ATAAATGGGT CTGGTTGGAA AAGAAGGAAA GCCTGGCTGA TCTGCTGGAG CTAGATTATT
7741 GCAGCAGGTA GGCAGGAGTT CCCTAGAGAA AAGTATGAGG GAATTACAGA AGAAAAACAG
7801 CACAAAATTG TAAATATTGG AAAAGGACCA CATCAGTGTA GTTACTAGCA GTAAGACAGA
7861 CAGGATGAAA AATAGTTTTG TAAACAGAAG TATCTAACTA CTTTACTCTG TTCATACACT
7921 ATGTAAAACC TACTAAGTAA TAAAACTAGA ATAACAACAT CTTTCTTTCT CTTTGTATTC
7981 AGTGTGGCAC ATCTGTAAAC GTTCACTCTT CACTTAGAGA CATCCTCAAC CAAATCACCA
8041 AACCAAATGA TGTTTATTCG TTCAGCCTTG CCAGTAGACT TTATGCTGAA GAGAGATACC
8101 CAATCCTGCC AGTAAGTTGC TCTAAAATCT GATCTGAGTG TATTTCCATG CCAAAGCTCT
8161 ACCATTCTGT AATGCAAAAA CAGTCAGAGT TCCACATGTT TCACTAAGAA AATTTCTTTT
8221 TCTCTTGTTT TTACAAATGA AAGAGAGGAC AAATAACATT TCTCTATCAC CGACCTGAAA
8281 CTCTACAGTC TTCAGAGAAT GAATGGCTTG CTAAAAGAAT GTCAAATCTT ACCATACAGC
8341 TATTTCATAT TACACTACTA AATACACTAT AAGGCATAGC ATGTAGTAAT ACACTGTAAA
8401 ATAGCTTTTT ACACTACTAT ATTATTAATA TCTGTTAATT CCAGTCTTGC ATTTCACATT
8461 TGCAAAACGT TTTGAAATTC GTATCTGAAA GCTGAATACT CTTGCTTTAC AGGAATACTT
8521 GCAGTGTGTG AAGGAACTGT ATAGAGGAGG CTTGGAACCT ATCAACTTTC AAACAGCTGC
8581 AGATCAAGCC AGAGAGCTCA TCAATTCCTG GGTAGAAAGT CAGACAAATG GTAAGGTAGA
8641 ACATGCTTTG TACATAGTGA GAGTTGGTTC ACCCTAATAC TGAGAACCTG GATATAGCTC
8701 AGCCAGCGTG CTTTGCGTTC AAGCTTACCA GAGCTGTTGT ATGCCTGTTA AGCAGGGCAT
8761 ACAGTCATGA GGCTCTTGAA AAATCTTAAC AGACAAAGGG CAATGGAAAA TCGGAGTTAA
8821 GGGATGGTAG GGATAAAATG CATAGAAAGA GGTACCACGA TTTTGATTTT TGCCCTAATG
8881 CCTCTCTGCG TGGTTCCTCA ATTTTTCTAC TTCATTCCTC ATCTCCTCAG AGCATTCCTT

8941 TCCCTCATGC TTGAAACACA GATGAAAGAC TGTGAATTCT AACTGAGATG AAAACATCCA
9001 CAACCACACA ACCTCTGGTG TGGAGTCACA TTCTGTGAAG GCAAAAACTA GGCCACGTAA
9061 TCTATGTGTG CAAGCTACGT GTAAGCTATG TGTGTGACAG GACAATGTGA GGAACATACT
9121 ATGTGCACAA GGACTGCAGA ATAAACAGGA GCAAAGTTTT TGAAGAAAAC AGAGTAAAAT
9181 CCCGTTTTCC TCTTTTGTTA CATTCTTTAC ATATATCTCA AATTTCCTCT TTGGTTAGAA
9241 GCAAGTAATA TTTATGTTTC TTGGTACTGT TTGGGTTGAA GACCATTCTG GGATAAGAGA
9301 AATTCCAGTG GTTCTTCCCC TAATCATAAA ATGTACAGGT TTAGTTTTTT TGTAACACAG
9361 AAATCTCTTC ATCTTTTATC TTTTGTTGTG ATTCTTTATA GAGAGAGAAA CAAGACTTAC
9421 TGACAATAGC AGCAAGAAAA TCAATCTTGG AAGAACAAGA TTGCAGTTGC AAAAACAAAC
9481 CAATGTCCTT GCCCCTACAT CCTCTTCCCC ATAAATTCTA CATTCTCTAT CTACCTTGTG
9541 CTTGCCAACA TGATATACGT AAACTCTCTT TTCCTATTCA TTCTTAAAGG AATTATCAGA
9601 AATGTCCTTC AGCCAAGCTC CGTGGATTCT CAAACTGCAA TGGTTCTGGT TAATGCCATT
9661 GTCTTCAAAG GACTGTGGGA GAAAGCATTT AAGGATGAAG ACACACAAGC AATGCCTTTC
9721 AGAGTGACTG AGGTATATGG GCATACCTTA GAGATGTAAT CTAGAATTTA TGAAGAGAGT
9781 AGACATGTTG TTATATGAAC ACTGCATTAG CGTATCTGCT CATTTGTCTG CATCTCTTTC
9841 AGACACTGTG TTAAAAGCAG GGAATTTTCC TTATGTCTCT TTCATCACAA TATTCCTGAC
9901 ATTGCAAAGC TCCTGAGAAA TAACTTCAGA TTCCCACTTT TCCTAGGGAG GTCTTCCTGG
9961 ATGAGAACAA TCAATCATCT TAACTGTAAC TAGATATTTC TGCATCTAAG AATAATCTTT
10021 GTTAAAACTA TATTCTCTCT CTCTTTTTTT TTTTTTTTGG TTCTCCAGCA AGAAAGCAAA
10081 CCTGTGCAGA TGATGTGGAT CC
